# Patients Discharged Against Medical Advice from a Psychiatric Hospital in Iran: A Prospective Study

**DOI:** 10.5539/gjhs.v6n3p213

**Published:** 2014-03-28

**Authors:** Fatemeh Sheikhmoonesi, Mohammad Khademloo, Samaneh Pazhuheshgar

**Affiliations:** 1 Psychiatry and Behavioral Sciences Research Center, Mazandaran University of Medical Sciences, Sari, Iran; 2 Department of Community Medicine, Mazandaran University of Medical Sciences, Sari, Iran; 3 Mazandaran University of Medical Sciences, Sari, Iran

**Keywords:** against medical advice (AMA), discharge, hospital, patient, psychiatry

## Abstract

**Aim:**

Self- discharged patients are at high risk for readmission and ultimately higher cost for care. We intended to find the proportion of patients who leave hospital against medical advice and explore some of their characteristics.

**Methods:**

This prospective study of discharge against medical advice was conducted in psychiatric wards of Zare hospital in Iran, 2011. A psychologist recorded some information on a checklist based on the documented information about the patient who wanted to leave against medical advice. The psychologist interviewed these patients and recorded the reasons for discharge against medical advice. Descriptive statistics were calculated for the variables.

**Results:**

The rate of premature discharge was 34.4%. Compared to patients with regular discharges, patients with premature discharge were significantly more likely to be male, self-employed, to have co morbid substance abuse and first admission and positive family history of psychiatric disorder. Disappearance of symptoms was the most frequent reason for premature discharge.

**Conclusion:**

The 34.4% rate of premature discharge observed in our study is higher than rate reported in other studies. One possible explanation is our teaching hospital serves a low-income urban area and most patients had low socioeconomic status. Further studies are needed to compare teaching and non-teaching hospital about the rate of premature discharge and the reasons of patients who want to leave against medical advice.

## 1. Introduction

Discharge against medical advice (AMA) is a prevalent and vexing problem for patients and their psychiatrists (Wung, CC Chen, FC Chen, & Lin, 2010). Some risks of AMA discharge include younger age, single marital status, male gender, low socioeconomic status, and combined diagnosis of personality or substance use disorder, lack of health insurance and history of numerous hospitalizations ending in AMA discharge (Brook, Hilty, Liu, Hu, & Frye, 2006; Said, Ibrahim, Kent, & Eswar, 2007).

Patients who leave hospital against medical advice may be at risk of adverse health outcome and readmission. Stephen, Hwang, Rajesh, Vince and Rochelle (2003) identified increased risk of readmission among general medicine patients who leave hospital AMA is concentrated in the first two weeks after discharge. Some reasons for AMA discharge are dissatisfaction with care, personal, family and financial problems and subjective improvement in symptoms (Said et al.,2007).

The problem in Iran is different from the West in many ways which is linked to cultural differences and the resources available for the care and treatment of mentally ill patients. We intended to find the proportion of patients who leave hospital against medical advice and some of their characteristics in the year 2011. Very little research in Iran has been conducted into AMA discharge from psychiatric hospitals. A drawback of the previous studies is that they are retrospective in nature and therefore susceptible to the bias of under-reporting of event. Moreover, they did not explore the reasons for AMA discharge.

As far as we know, this study is the first prospective study in Iran which was carried out through interviews with people who were willing to have AMA discharge. The reasons of AMA discharge are the main concerns of this study. Awareness of the factors involved in AMA discharge is important because of the potential to identify those at higher risk and thereby intervene earlier to prevent adverse consequences.

## 2. Materials and Methods

### 2.1 Subjects and Data Collection

This prospective study of AMA discharge was conducted in psychiatric wards of Zare hospital, a major psychiatric center in the north of Iran. AMA discharge was defined as “patients who signed a standard hospital form acknowledging that they were discharging themselves against medical advice contrary to the advice of their psychiatrist”.

### 2.2 Participants Recruitment

Three hundred and sixteen patients who were discharged between 1 January 2011 and 1 January 2012 were randomly assessed. We use simple randomization to choose patients to be interviewed. As soon as AMA discharge was reported by nurses, a psychologist recorded some information on a checklist based on the documented information about the patient including socio-demographic variables, diagnosis according to DSM-IV-TR (Diagnostic and statistical Manual of Mental disorders, 4th edition; American Psychiatric Association, 1994), patients and their caregiver`s level of education, substance abuse, number of previous hospitalizations, and family history of psychiatric disorder. The psychologist interviewed the patients and recorded the reasons for AMA discharge on a checklist. More than one reason could be coded for each patient. The data was collected and then analyzed anonymously.

### 2.3 Statistical Analysis

Descriptive statistics were calculated for the variables. X2 test and independent t-test were applied to compare the variables between patients with AMA discharges and those with regular discharges. The data was analyzed using SPSS version 10.0 for windows.

### 2.4 Ethical Issues

This study was approved by the Ethical Committee of Mazandaran University of Medical Sciences. All the patients signed informed consents.

## 3. Results

Of 1171 patients discharged during the study period, 403 patients were discharged AMA. The rate of AMA discharge was 34.4% according to Molnar & Pinchoff equation (1993). Three hundred and sixteen patients were randomly assessed. Among the completed questionnaires there were 9 questionnaires with no information on profession and 6 of them did not provide the information on marital status. However, we included these questionnaires since they contained other information.

One hundred and thirty two (41.77%) were male and 184 (58.22%) were female. The mean age was 35.1±11.93 and 226 (71.51%) were discharged as planned but 90 patients (28.48%) were discharged AMA. Compared to patients with regular discharges, AMA discharge patients were significant differences between the groups regarding the age, marital status, educational level, previous suicide attempt. Table 1 summarizes the baseline characteristics of the study sample. AMA discharge was more prevalent in significantly more likely to be male, self-employed, to have co morbid substance abuse and first admission and positive family history of psychiatric disorder. There were no patients admitted for the first time (P=0.004).

**Table 1:** Characteristics of patients who left hospital AMA and patients discharged formally

Characteristic	Left AMA (n=90)	Discharged formally (n=226)	Pvalue
**Sex**			0.034
Male	46(34.8%)	86(65.2%)	
Female	44(23.9%)	140(76.1%)	
**Mean age**	33.7±12	35.63±11.8	NS
**Profession**			
Unemployed/housewife	59(65.6 %)	187(82.8%)	
Self-employed	20(22.2%)	23(10.17%)	0.006
Office worker	7(7.8%)	11(4.82%)	
Missing data	4(4.4%)	5(2.21%)	
**Marital Status**			NS
single	31(34.4%)	93(41.1%)	
Married	41(45.6 %)	90(40%)	
Widow	14(15.6 %)	32(14%)	
Divorced	0(0%)	9(4%)	
Missing data	4(4.4%)	2(0.9%)	
**Educational level**			
elementary school	27(30%)	69(30.5%)	NS
High school	47(52%)	98(43.4%)	
college	16(18%)	59(26.1%)	
**First Admission**	65(65%)	96(42%)	0.001
**Positive family history**	26(21.3%)	96(78.7%)	0.029
**History of substance abuse**	38(42.2%)	75(33.1%)	0.05
**History of suicide attempt**	24(26.7%)	36(16%)	NS

NS: not significant statistically.

Among the patients the educational level of 96 was elementary school of whom 28.1% were discharged against medical advice and 71.9% were formally discharged. There were 145 patients with educational background at high school level. Amongst them 32.4% had AMA discharge but 67.6% had regular discharge. From the total of 75 patients with college education 21.3% left the hospital AMA while 78.7% were discharged formally. These figures indicate no significant difference between the two groups regarding educational background.

There were no significant differences between the groups regarding season of admission, the data is presented in Table 2.

**Table 2:** Season of admission in two groups

Season	Left AMA (n=90)	Discharged formally (n=226)	Pvalue
**Spring**	16(17%)	55(25%)	0.161
**Summer**	26(29%)	52(23%)	
**Autumn**	6(7%)	29(13%)	
**Winter**	42(47%)	89(39%)	

Disappearance of symptoms was the most frequent reason for AMA discharge (Table 3). As shown in Table 4, there was no significant difference between the groups with respect to diagnosis according to DSM-IV-TR.

**Table 3:** Reasons for AMA discharge from psychiatric ward

Reasons for AMA discharge	Frequency
**Disappearance of symptoms**	32(35.6%)
**Lack of insight**	23(25.6%)
**Missing family**	17(18.9%)
**Boredom from ward environment**	15(16.7%)
**Economic problem**	11(12.2%)
**Fear of other patients**	9(10%)
**Anger over given treatment**	8(8.9%)
**Lack of trust to the doctor**	3(3.3%)
**Lack of satisfaction with staff behavior**	4(4.4%)
**Others**	8(8.9%)

**Table 4:** Comparison of diagnosis in two groups

	Diagnosis Left AMA (n=90)	Discharged formally (n=226)
**MDD**	13(41.9%)	18(58.1%)
**Bipolar disorder (type I, II)**	26(25.7%)	75(74.35%)
**Psychotic disorder**	44(27%)	119(73%)
**Anxiety Disorder**	7(33.3%)	14(66.7%)
**Personality Disorder**	5(25%)	15(75%)

## 4. Discussion

Self-discharged patients are at high risk for re-admission and ultimately higher costs of care (Anis et al.,2002). These patients are also at high risk for poor follow-up care for medical issues. Understanding the characteristics of patients who leave AMA and gaining insight into their reasons are great importance since delivery of medical care and quality of care are affected (Said et al.,2007). Prospective nature of this study with the opportunity to interview the patients and investigate their reasons to AMA discharge was an advantage of this study. We found that 34.4% of psychiatric hospitalization ended in patients leaving against medical advice. The 34.4% rate of discharge AMA observed in our study is higher than other rate reported in previous studies (Tavallaei et al., 2006; Said et al., 2007; Wung et al.,2010) one possible explanation is our hospital serves a low-income urban area and most patients had low socioeconomic status.

Weingart, Davis and Phillips (1998) reported male gender to be associated with AMA discharge. Our observations and those of others (Saitz et al., 1999; Jeremiah, O’Sullivan, & Stein, 1995) confirm these findings. Perhaps men are willing to come back home earlier for economic reasons since they feel most responsible for their family’s income. Another possible explanation contributing to AMA discharges is that male patients are more likely to have a higher risk of poor medical adherence than female patients (Nose, Barbui, & Tansella, 2003), poor medical adherence leads to premature discharge consequently (Wung et al.,2010).

Patients with positive family history are less likely to leave against medical advice. One explanation might be that caregivers are more aware of psychiatric disorders and feel worried of not being able to provide better care at home.

Data analysis revealed more AMA discharge amongst self-employed, probably because they were more worried about their jobs while other patients such as office workers had the advantage of sick leave.

We found that the rate of AMA discharge was more prevalent among patients hospitalized for the first time, this may be due to unfamiliarity of the patient or his caregiver with hospital environment thereby resisting hospitalization. In addition they are not aware of the course and probability of recurrence in psychiatric disorder.

Data analysis showed substance abuse as a risk factor for irregular discharge. There are some studies in support of this finding that substance abuse could increase the risk of AMA discharge (Gillis, Russell, & Busby, 1997; Glick, Braff, Johnson, & Showstack, 1981; Ahmadi et al., 2006; Saitz et al., 2002). Perhaps patients with substance abuse are unwilling to stay in hospital, as hospital stay makes it difficult for them to obtain the required substance. Chan, Palepu and Guh (2004) found that AMA discharges were less likely among injection drug abusers if they were receiving methadone in the hospital. Protocols to treat alcohol and opioid withdrawal to reduce AMA discharge have not been studied yet. Given the high prevalence of AMA discharges in these patients; this would be a worthy area of studying.

Data analysis showed that there was not any significant difference between two groups with respect to diagnosis but Brook et al. (2006) found antisocial personality disorder to be associated more with AMA discharge.

In our study, AMA discharge was not different among different seasons. The authors carried out a study about patients absconding from Zare hospital. In that study we found absconding was more frequent in summer (Sheikhmoonesi, Kabirzadeh, Yahyavi, & Mohseni, 2012).

We found that age and educational level were not the risk factor for irregular discharge. This conclusion is similar with those found in other studies carried out in Iran (Ahmadi et al., 2006). In most studies conducted in developed countries younger patients and those with low level of education most frequently discharged against medical advice (Senior & Kabbee, 1986; Ferber et al.,1985). It is difficult to explain the reason, nevertheless it could depends on cultural factors. Smith (1982) proposed that differences in hospital setting, client population and therapists’ variety might be the cause of differences between the risk factors of a premature discharge.

Interviewing the patients in order to find the reasons for AMA discharge was done and the results of this study have shown disappearance of symptoms (feeling better) and lack of insight as the main reasons. The tolerance to hospitalization reduces if a person does not believe in his/her disease so proper orientation and psycho educational session during the early treatment phase is essential to keep the patient in hospital.

Targum, Capodanno, Hoffman and Foudraine (1982) found an approximately 30% decrease in total AMA discharges among psychiatric inpatients that used a nurse as a patient advocate. The advocate’s responsibility was to help explore a patient’s preconceptions about hospitalization and to address fears and complaints about it.

Limitation: Our results should be interpreted with several limitations in mind. The length of stay at hospital and its relationship with AMA discharge was not assessed. Our study was done in a teaching hospital so we cannot generalize our findings to other hospitals. Larger studies are needed to compare teaching and non-teaching hospitals about the rate of AMA discharge and the reasons of patients who want to leave AMA.

## 5. Conclusion

Awareness of the factors involved in AMA discharge is important because of the potential to identify those at higher risk and thereby intervene earlier to prevent adverse consequences. Male gender, self-employed, to have co morbid substance abuse and previous hospitalizations and positive family history of psychiatric disorder are risk factors to discharge against medical advice.
